# Group A *Streptococcus* Meningitis in Adults, Denmark

**DOI:** 10.3201/eid2909.230627

**Published:** 2023-09

**Authors:** Henrik Nielsen, Merete Storgaard, Jannik Helweg-Larsen, Lykke Larsen, Micha P.G. Jepsen, Birgitte R. Hansen, Lothar Wiese, Jacob Bodilsen

**Affiliations:** Aalborg University Hospital, Aalborg, Denmark (H. Nielsen, J. Bodilsen);; Aalborg University, Aalborg (H. Nielsen, J. Bodilsen);; Aarhus University Hospital, Aarhus, Denmark (M. Storgaard);; Rigshospitalet, Copenhagen, Denmark (J. Helweg-Larsen);; Odense University Hospital, Odense, Denmark (L. Larsen);; Nordsjællands Hospital, Hillerød, Denmark (M.P.G. Jepsen);; Hvidovre University Hospital, Hvidovre, Denmark (B.R. Hansen);; Zealand University Hospital, Roskilde, Denmark (L. Wiese)

**Keywords:** group A Streptococcus, meningitis/encephalitis, epidemiology, bacteria, Denmark

## Abstract

We report a 21-fold increase in group A *Streptococcus* meningitis in adults in Denmark during October 13, 2022–April 12, 2023, concurrent with an outbreak of invasive streptococcal disease. We describe clinical characteristics of the outbreak cases and prognosis for patients in comparison to those for previous sporadic cases.

Emergence of increased group A *Streptococcus* (GAS) disease, initially expressed as activity of scarlet fever in childhood, has been observed in multiple countries; some countries reported the toxigenic M1_UK_ clone (*1*–*3*). A report from the Netherlands suggested an increase in GAS meningitis cases, mainly from the toxicogenic M1_UK_ lineage (*4*). This increase is likely result of the rise in invasive GAS infections (*5*), because ≈1% of invasive GAS manifests as meningitis (*6*). However, it is unclear if this outbreak differs clinically from previous sporadic cases, as acknowledged by van der Putten et al. (*4*). To address this limitation, we compared all cases of GAS meningitis in adults in Denmark during 2015–2022 with cases during the outbreak, October 2022–April 2023.

The Danish Study Group for Infections of the Brain (DASGIB) has performed active, real-time nationwide surveillance of community-acquired bacterial meningitis in adults (>18 years of age) since January 1, 2015, as described previously (*7*). In brief, data on demographics, comorbidities, clinical signs and symptoms, microbiology and biochemical examinations, radiology, treatment, and outcome are aggregated in an online platform. The legal department of the North Denmark Region (record no. 2023-012693) and the Danish Board of Health (record nos. 3-3013-2579/1 and 3-3013-3168/1) approved the DASGIB database. Patient consent or permission from an ethical committee is not required.

For this study, a definition of GAS meningitis required (*7*) clinical symptoms suggestive of bacterial meningitis (e.g., headache, neck stiffness, fever, altered mental status) and either of the following criteria: positive culture or bacterial DNA/antigen analysis of cerebrospinal fluid (CSF); positive blood culture and CSF leukocytes >10 × 10^6^ cells/L; or culture–confirmed otitis or mastoiditis and CSF leukocytes >10 × 10^6^ cells/L. Incidence was computed as no. cases/no. adults in Denmark during each study period.

During January 1, 2015–October 12, 2022, we observed a total of 8 cases of GAS meningitis, corresponding to a mean of 0.11/1 million adults/6 months (Figure). Because of the increase in invasive GAS in Denmark beginning in October 2022 (*8*), we then assessed the incidence of GAS meningitis during October 13, 2022–April 12, 2023. We observed 11 cases of GAS meningitis in adults, corresponding to 2.32/1 million/6 months, an increase in incidence by a factor of 21. The diagnosis was confirmed by culture in 9 patients, whereas it was established by PCR in 2 patients for whom antimicrobial treatment began before lumbar puncture. We examined isolates of *emm*-1.0 type in 4 cases, *emm*-12.0 in 2 cases, and *emm*-87.0 in 1 case; isolate type was not available in 2 cases.

**Figure:**
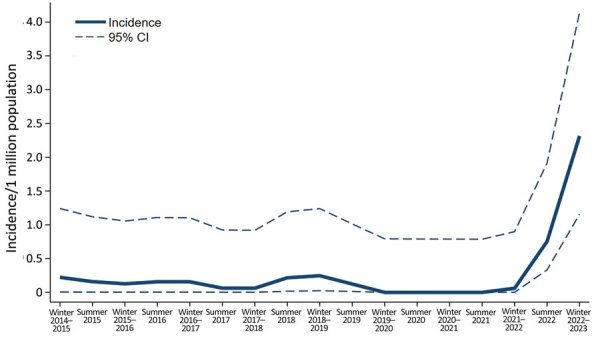
Incidence of community-acquired group A Streptococcus meningitis in adults in winter periods (October–March) and summer periods (April–September), Denmark, January 1, 2015–2023, illustrating outbreak during October 13, 2022–April 12, 2023.

Patients with GAS meningitis had lower Glasgow Coma Scale scores at admission and higher CSF leukocyte counts in the last 6 months of the study than overall (Table); otherwise, clinical characteristics and prognosis did not differ between the 2 study periods. We observed a high percentage of patients with streptococcal infection in the upper respiratory tract (Table). We observed 2 serious complications, endophthalmitis (1 case) and subdural empyema (1 case), but no increase in deaths in the second study period.

**Table:** Characteristics of adults with community-acquired group A streptococcal meningitis, Denmark, 2015–2023

Characteristic	Jan 1, 2015–Oct 12, 2022	Oct 13, 2022–Apr 13, 2023	p value†
Total no. cases	8	11	
Age	57 (35–66)	58 (40–69)	0.53
Sex, no. (%)			
M	5 (62)	7 (64)	
F	3 (38)	4 (36)	1.0
Comorbidity, no. (%)	0	4 (36)	0.09
Duration of symptoms, d	5.5 (1–7)	2 (1–4)	0.35
Glasgow Coma Score‡	15 (15–15)	13 (11–15)	0.01
Temperature	37.7 (37.0–39.0)	39.1 (37.7–39.4)	0.23
Systolic blood pressure	131 (113–136)	125 (119–125)	0.25
Ear-nose-throat focus, no. (%)	7 (88)	9 (82)	1.0
C-reactive protein, mg/L	305 (228–367)	216 (105–313)	0.46
Time until lumbar puncture, h	3.0 (1.8–3.6)	2.2 (1.6–7.8)	0.85
CSF leukocytes, 10^6^ cells/L	111 (40–385)	1,726 (534–3,990)	0.03
CSF protein, g/L	1.7 (0.7–3.2)	1.6 (0.9–2.6)	0.79
CSF culture positive, no. (%)	3 (38)	3 (27)	1.0
Bacteremia, no. (%)	5 (63)	7 (64)	1.0
Time until antimicrobial drugs, h	3.7 (1.7–7.0)	4.4 (0.3–12.3)	0.95
Dexamethasone, no. (%)	8 (100)	8 (73)	0.23
Intensive care unit stay, no. (%)	4 (50)	7 (64)	0.66
Progressive or new neurologic deficits, no. (%)	3 (38)	1 (9)	0.26
Seizures, no. (%)	2 (25)	1 (9)	0.55
Septic shock, no. (%)	3 (38)	1 (9)	0.26
Death, no. (%)	1 (13)	1 (9)	1.0

*Values are median (IQR) except as indicated. CSF, cerebrospinal fluid; IQR, interquartile range. †p value determined by Fisher exact test for categorial variables and Mann-Whitney rank sum test for continuous variables. ‡Glasgow Coma Score based on eye opening (*1*–*4*), verbal response (*1*–*5*) and motor response (*1*–*6*), maximum 15 points.

We conclude that in October 2022–April 2023, an outbreak of GAS meningitis occurred in Denmark, showing a 21-fold increase in incidence compared with the baseline in previous years. The baseline incidence agrees with earlier findings in Denmark (*9*). Our case definition included cases confirmed by positive PCR of CSF, positive blood cultures or other cultures combined with CSF pleocytosis, and clinical manifestations of bacterial meningitis, in addition to positive CSF culture, which may explain why our incidence is higher than that recently reported for adults from the Netherlands (*4*).

The rise in invasive GAS infections was initially seen in children (*5*), but our study indicates an increase of severe infections in adults as well. The toxicogenic *emm*-1.0 type is currently the predominant strain in Denmark (*8*) and other countries (*4*,*5*). However, we found no differences in clinical characteristics or prognosis for GAS meningitis during this surge compared with those of previous years.
